# Advances in actinomycete research: an ActinoBase review of 2019

**DOI:** 10.1099/mic.0.000944

**Published:** 2020-06-19

**Authors:** Samuel M.M. Prudence, Emily Addington, Laia Castaño-Espriu, David R. Mark, Linamaría Pintor-Escobar, Alicia H. Russell, Thomas C. McLean

**Affiliations:** ^1^​ School of Biological Sciences, University of East Anglia, Norwich Research Park, Norwich, Norfolk NR4 7TJ, UK; ^2^​ Strathclyde Institute of Pharmacy and Biomedical Sciences, University of Strathclyde, 161 Cathedral Street, Glasgow, G4 0RE, UK; ^3^​ Department of Biology, Edge Hill University, Lancashire, L39 4QP, UK; ^4^​ Department of Molecular Microbiology, John Innes Centre, Norwich, NR4 7UH, UK

**Keywords:** ActinoBase, *Actinobacteria*, antibiotics, development, methodology, microbial ecology, regulation, specialized metabolites, *Streptomyces*, symbiosis

## Abstract

The actinomycetes are Gram-positive bacteria belonging to the order *Actinomycetales* within the phylum *Actinobacteria *. They include members with significant economic and medical importance, for example filamentous actinomycetes such as *Streptomyces* species, which have a propensity to produce a plethora of bioactive secondary metabolites and form symbioses with higher organisms, such as plants and insects. Studying these bacteria is challenging, but also fascinating and very rewarding. As a Microbiology Society initiative, members of the actinomycete research community have been developing a Wikipedia-style resource, called ActinoBase, the purpose of which is to aid in the study of these filamentous bacteria. This review will highlight 10 publications from 2019 that have been of special interest to the ActinoBase community, covering 4 major components of actinomycete research: (i) development and regulation; (ii) specialized metabolites; (iii) ecology and host interactions; and (iv) technology and methodology.

## Introduction

**Fig. 1.**
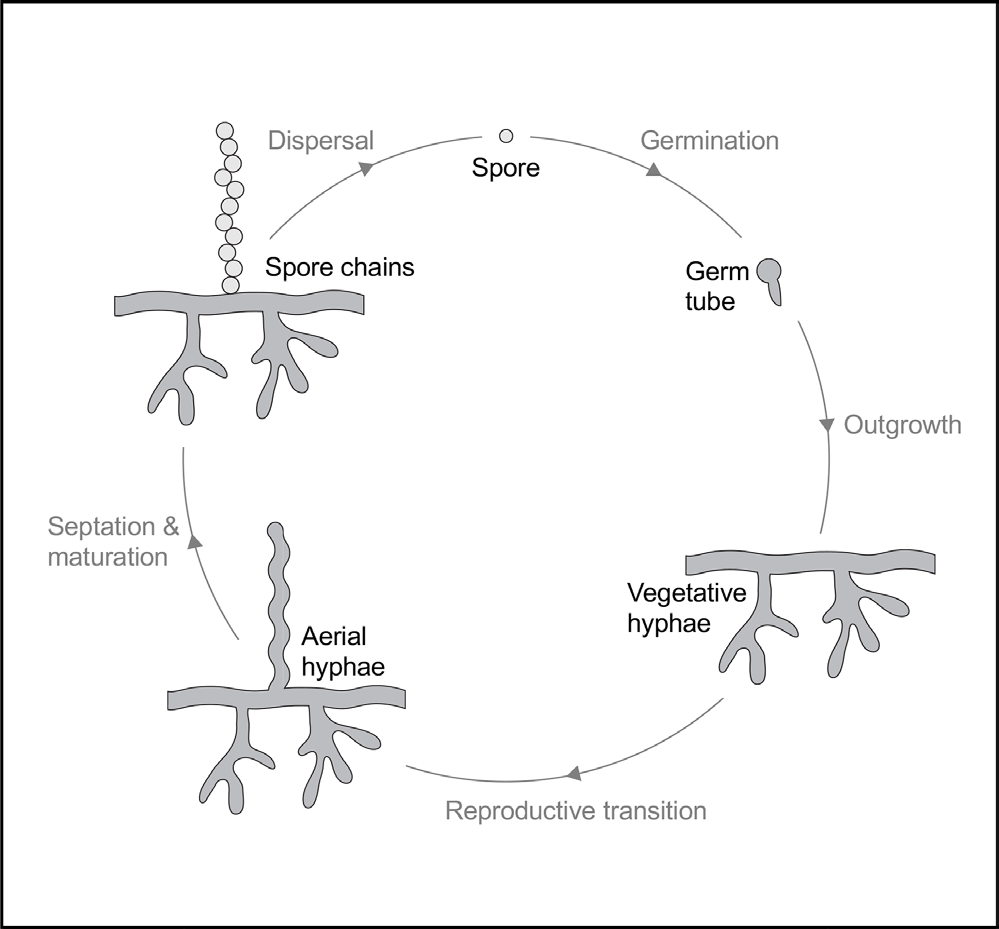
The life cycle of filamentous actinomycetes based upon the model organism *S. venezuelae *. Development begins with the formation of one or two germ tubes from a spore. Following germination, mycelial outgrowth leads to the formation of vegetative mycelium. Transition from vegetative growth to aerial hyphae production occurs upon detection of signals, e.g. nutrient depletion. The aerial hyphae septate and mature into chains of pigmented spores that disperse, and the cycle can then begin again.

The order *Actinomycetales* (commonly referred to as actinomycetes) is a group within the Gram-positive phylum *Actinobacteria* containing genera of filamentous bacteria such as *Amycolatopsis *, *Micromonospora, Pseudonocardia*, *Saccharopolyspora* and *Streptomyces* species and unicellular bacteria such as *Corynebacterium* and *Mycobacterium* species [1]. The name actinomycete derives from the ancient Greek *ἀκτίς (aktís,* ‘ray’) and *μύκης (múkēs,* ‘mushroom or fungus’) after the mycelium formation and hyphal tip extension-driven growth [2, 3]. Members of the order *Actinomycetales* show considerable physiological diversity. Most actinomycetes are aerobic, saprophytic micro-organisms with complex lifecycles (Fig. 1) but, as always, exceptions frequently occur [4].

Actinomycetes are ubiquitous in the environment and are capable of both solitary inhabitation and forming symbioses, not only with other micro-organisms but also higher-order creatures [5]. In addition to this actinomycetes, and in particular members of the genus *Streptomyces,* are prolific producers of specialized metabolites. These bacteria have proven a bountiful source of bioactive chemicals, contributing two-thirds of the clinically applicable antibiotics and a wide range of industrially important enzymes [6]. Biosynthesis of these natural products is intrinsically linked to the organisms’ complex developmental life cycle, with the majority of production in *Streptomyces* species coinciding with the transition from vegetative to aerial hyphal growth. This progression can occur due to numerous factors, including nutrient deprivation, abiotic stress or microbial competition, requiring complex regulatory systems to control development and specialized metabolite biosynthesis [7].

However, research on actinomycetes can be challenging due to a few key attributes. Developmental cycles can last weeks or months, prolonging common research practices. The GC-rich genome impedes routine molecular techniques, whilst abundant repeat regions complicates genome sequencing and assembly [8, 9]. In general, there is a scarcity of specialized tools and techniques for non-model organisms, frustrating research efforts.

ActinoBase (https://actinobase.org) is a Microbiology Society-supported initiative that has been launched with the aim of developing and distributing knowledge and resources pertaining to the study of filamentous actinomycetes, built by the research community for new and existing actinomycete researchers. Beyond the collation of information, it is the basis of a drive to connect the global filamentous actinomycete research community, which meets approximately every 3 years at the International Symposium on the Biology of Actinomycetes (ISBA). ActinoBase purposefully excludes the *Mycobacteriaceae *, as researchers on this bacterial family have already developed numerous specialized tools and resources within their own community [10].

To exemplify the four main avenues of filamentous actinomycete research, this review has been split into four sections: (i) development and regulation; (ii) specialized metabolites; (iii) ecology and host interactions; and (iv) technology and methodology. The purpose of this review is to feature publications that we, the early-career researchers who have established ActinoBase, believe to be of special interest to the actinomycete research community. We do not intend to highlight the ‘top’ papers of 2019 by any metric. We hope to use this opportunity as a lens through which we can highlight the breadth and intrigue contained within the actinomycete research field, and to provide an overview of what studying these organisms can entail.

### Development and regulation

Streptomycetes possess a fascinating and complex developmental life cycle that links cell differentiation with specialized metabolism. These morphological and metabolic progressions require tight, multi-layered regulation to detect external and internal stimuli and thus trigger cellular responses. As part of the ongoing interest in the regulatory networks of streptomycetes, 2019 saw new reviews on different signal transduction mechanisms in this genus. McLean and colleagues [11] screened the genomes of 93 fully sequenced streptomycetes for two-component systems (TCSs) and found 15 that were conserved across all species. Their analysis of recent literature revolved around these 15 conserved TCSs plus 7 others of interest and highlighted the rapid progress in this area in recent years. For another signalling mechanism, Latoscha *et al.* [12] compiled information on the four nucleotide secondary messengers known to date in *Streptomyces* species – (p)ppGpp, cAMP, c-di-AMP and 3′,5′-cyclic diguanylic acid (c-di-GMP) – and summarized findings on the enzymes responsible for their synthesis and degradation, the effector molecules and the subsequent cellular response triggered by these signalling cascades.

#### c-di-GMP arms an anti-σ to control progression of multicellular differentiation in *Streptomyces* [13]

In line with the review by Latoscha *et al.* [12], a seminal study by Gallagher and colleagues [13] demonstrated that a secondary messenger, c-di-GMP, plays an essential role during sporulation in *Streptomyces* species.

During the later stages of development, the sporulation-specific σ^WhiG^ plays a key role in controlling the differentiation of aerial hyphae into spores. To investigate the role played by σ^WhiG^, Gallagher *et al.* deleted *whiG* in *Streptomyces venezuelae *, which resulted in an oligosporogenous phenotype. Conversely, overexpression of the σ factor resulted in hypersporulation. Combining chromatin immunoprecipitation sequencing (ChIP-seq) with transcriptional profiling, it was revealed that σ^WhiG^ controlled the expression of *whiH*, *whiI* and *vnz_15005.* The first two of these genes are late developmental genes, whilst *vnz_15005* encodes for a membrane protein of unknown function [13]. The authors also identified the corresponding anti-σ factor to σ^WhiG^, RsiG, which was shown to form a direct complex together and regulates σ^WhiG^ activity. Interestingly, RsiG showed no homology to any previously identified anti-σ factor, so the mechanism of binding was unclear. To address this, Gallagher *et al.* determined the crystal structures of the complex, revealing a 1 : 1 ratio of RsiG to σ^WhiG^. The structures also revealed that the secondary messenger, c-di-GMP, was key to the complex by linking the two proteins together. Further, c-di-GMP was found to be in a novel symmetrical dimeric form, mediating not only contact, but also correct forming of the RsiG loop around σ^WhiG^. Finally, fluorescent polarization-based binding experiments revealed that RsiG discriminates between c-di-GMP and closely related molecules, including c-di-AMP, with key arginine and aspartic acid residues controlling this interaction [13].

Whilst previous work had established the link between c-di-GMP and the master regulator of aerial hyphae formation (BldD, 14), the results of Gallagher and colleagues’ study confirm that c-di-GMP is not only a key modulator of the last stages of the developmental progression, but also a central repressor of development in *Streptomyces* species. These findings support the results of two other papers published in 2019 that explore the role of enzymes involved in the synthesis of c-di-GMP in the developmental programme of streptomycetes [15] and the effect of overexpressing them to increase the yield of erythromycin in *Saccharopolyspora erythraea* [16].

Gallagher and colleagues’ work also identified a new class of anti-σ factor and described the first example of c-di-GMP targeting a σ–anti-σ factor pair in *Streptomyces* species. However, the question remains as to what exactly the activating signal is for the production and degradation of nucleotide second messengers such as c-di-GMP [13].

#### Silencing of specialized metabolism in *Streptomyces* by the nucleoid-associated protein Lsr2 [17]

*Streptomyces* biosynthetic gene clusters (BGCs) encoding the production of specialized metabolites are activated by pathway-situated regulators that are controlled by transcription factors. However, only a small fraction of BGCs are transcribed under normal laboratory conditions [18], resulting in a bottleneck in the specialized metabolites discovery process. The roles of several pathway-situated regulators in specialized metabolite production have been previously identified. For example, the production of actinorhodin in *Streptomyces coelicolor* is regulated by ActII-4 [19] and CprB [20]. There are also an increasing number of examples of coordinate regulation from one cluster-situated regulator on another BGC [21], such as with FscRI regulating both candicidin and antimycin production in *Streptomyces albidoflavus* S4 [22]. Lsr2 is a global transcriptional regulator conserved throughout the phylum *Actinobacteria* that plays an important role in the repression of gene expression. However, until now, Lsr2 has mainly been studied in *Mycobacterium* spp. [23].

Gehrke *et al.* report that all streptomycetes encode two nucleoid-associated proteins, Lsr2 and Lsr2-like (LsrL), and that these are involved in *Streptomyces* gene regulation. The authors also show that Lsr2 plays an important role in secondary metabolism. A total of 223 Lsr2-binding sites were found distributed throughout the *S. venezuelae* chromosome (mainly across the core region) and it was shown that Lsr2 binds DNA to repress gene expression. Furthermore, it was demonstrated that Lsr2 plays a major role in repressing the gene expression from sequences with significantly higher AT content [17].

The biological roles of Lsr2 and LsrL were studied in four *S. venezuelae* strains; the wild-type strain alongside *Δlsr2*, *ΔlsrL* and ∆*lsr2∆lsrL* mutant strains. It was found that deleting *lsrL* and *lsr2* from the chromosome of *S. venezuelae* had minor effects on development. However, deleting *lsr2* significantly affected *S. venezuelae *’s specialized metabolism, including a stark increase in melanin production compared to the wild-type [17]. A total of 484 genes displayed altered expression in the *lsr2* mutant strain when compared to the wild-type strain and the expression of 90 % of these genes was upregulated. It was observed that Lsr2 is directly involved in secondary metabolism of *S. venezuelae* by repressing the transcription of cryptic BGCs. Of the 484 genes with altered expression, 155 correlated with BGCs that encode the production of specialized metabolites [17]. For example, it was observed that the increased melanin production correlated with an increased expression of genes within the melanin biosynthetic gene cluster. As reported in the paper, these results suggest that Lsr2 represses the activity of both well-conserved and newly acquired BGCs under normal laboratory conditions, explaining the ‘silencing’ of many BGCs and their ‘awakening’ under specific conditions [17].

Further liquid chromatography/mass spectrometry (LC-MS) analysis revealed that the four different strains used in this study produced a strain-specific chemical profile. It was observed that the *lsr2* deletion significantly increased the production of specialized metabolites when compared to the wild-type, many of which were novel. Furthermore, *lsr2* deletion promoted the production of otherwise cryptic metabolites in a wide range of *Streptomyces* species. In summary, deletion of *lsr2* upregulated cryptic BGCs, which enabled the production of new specialized metabolites [17]. Together with previous reports [24], this study demonstrated the importance of deleting the global regulatory gene *lsr2* to induce novel antibiotic production in *Streptomyces* species [17].

These featured publications highlight the complexity of actinomycete regulatory systems in both development and secondary metabolism. As has been highlighted above, the ability to perturb development and regulatory elements has repeatedly led to the production of novel specialized metabolites. However, the exploitation of this can be greatly improved with a better understanding of the basic underlying mechanisms of regulation, an area where more research is dearly required.

### Specialized metabolites

Advances in microbial genome sequencing have revealed that bacteria are capable of producing many more natural products than are currently characterized, and actinomycetes such as *Streptomyces* species are particularly talented producers of secondary metabolites [25, 26]. Whilst many of these compounds are antimicrobial, they can also be anticancer, antiviral or immunosuppressive in activity. However, identifying novel molecules remains a challenge. Traditional activity-based screening of microbial extracts often results in the rediscovery of already known compounds, and therefore novel approaches to natural product discovery are now required [27].

#### Salinipeptins: integrated genomic and chemical approaches reveal unusual d-amino acid-containing ribosomally synthesized and post-translationally modified peptides (RiPPs) from a Great Salt Lake *Streptomyces* sp. [28]

One exciting avenue of natural product research is the isolation of bacteria from unique environments in order to study their biosynthetic potential. A study by Shang *et al.* achieved this by isolating several actinomycetes from the hypersaline environment of the Great Salt Lake. Interestingly, these isolates were closely related to bacteria that have been identified from marine environments, based on 16S rRNA gene sequencing. The genome of one of these halotolerant isolates, *Streptomyces* sp. GSL-6C, was sequenced and analysed, leading to the identification of a BGC encoding for production of linaridins, a rare subfamily of ribosomally synthesized and post-translationally modified peptides (RiPPs, 26).

RiPPs are a structurally diverse class of natural products that display a range of important bioactivities such as antimicrobial and anticancer activity. These compounds are produced from a small precursor peptide that is synthesized on the ribosome and post-translationally modified by a series of tailoring enzymes [29]. Intriguingly, RiPP BGCs have historically been difficult to detect with genome mining, and recent studies are starting to reveal the vast untapped biochemical diversity of these compounds [30, 31].

The linaridin RiPP subfamily comprises only three characterized examples: cypemycin [32], grisemycin [33] and legonaridin [34]. Among other post-translational modifications (PTMs), these compounds feature rare N-terminal methylations that are essential for their antibiotic activity [35].

*Streptomyces* sp. GSL-6C was subjected to large-scale cultivation in saline nutrient medium in order to isolate the peptides produced by the identified BGC. This led to the isolation of four modified peptides, named salinipeptides. Nuclear magnetic resonance (NMR) spectra for the major peptide, salinipeptide A, suggested that the peptide had undergone significant modifications. A combination of genetic information, NMR and mass spectrometry data allowed validation of the amino acid sequence, which included 15 proteinogenic and 6 nonproteinogenic amino acids. The absolute configurations of the amino acids were confirmed with an advanced Marfey’s analysis, which revealed that several of the residues were of d-configuration. The salinipeptides were the first reported members of linaridins to harbour d-amino acids and, furthermore, d-proline residues have not been reported for any known RiPPs. Further analysis into the possible mechanism of amino acid isomerization suggested that novel enzymology may be involved in the generation of salinipeptide d-amino acids, as homologues of known isomerases could not be identified in the *Streptomyces* sp. GSL-6C genome [28]. As well as amino acid reconfiguration, additional PTMs in salinipeptide A include formation of an aminovinyl-cysteine end group and dehydration of threonine residues to dehydrobutyrine (Dhb). Salinipeptide A undergoes N-terminal oxidation to yield salinipeptide B, followed by proposed oxidation, dehydration or elimination and cyclization to yield salinipeptide C. Salinipeptide D only differs from A by the presence of one threonine residue that is not dehydrated to Dhb. As well as interesting structural features, salinipeptide A also displayed some antibacterial activity against *Streptococcus pyogenes* [28].

Overall, this work by Shang *et al.* shows how integrated genomic and chemical analyses can be applied to uncover novel natural products from actinomycetes isolated from unique environmental niches, approaches that could be crucial to uncovering new antibiotics in the future. Furthermore, identification of novel enzymology helps to shed light on the complex biosynthetic machineries employed in nature to produce structurally diverse molecules.

#### Characterization of the mode of action of aurodox, a type III secretion system inhibitor from *Streptomyces goldiniensis* [36]

Work published early in the year showed the potential of identifying novel roles for previously shelved natural products, with McHugh *et al.* characterizing the anti-virulence activity of aurodox [36]. First purified from *S. goldiniensis* in the 1970s, aurodox was shown to possess only weak antimicrobial activity from its ability to bind elongation factor Tu and inhibit protein synthesis [37], and so the molecule never made it into clinical use. In 2011 aurodox was identified as a potent inhibitor of the type III secretion system (T3SS) in enteropathogenic *Escherichia coli* (EPEC) and was able to protect mice from T3SS-mediated pathogenesis from *Citrobacter rodentium* [38], sparking interest in developing the molecule as a candidate anti-virulence drug. In their work, McHugh *et al.* identified the mechanism by which aurodox is able to inhibit the T3SS. Even at the highest concentrations, 5 µg ml^−1^, aurodox was unable to inhibit EPEC or *C. rodentium* cell viability. This suggested that T3SS was inhibited independently of any growth defects. Using an *in vitro* infection assay, it was discovered that aurodox inhibits the ability of enterohemorrhagic *E. coli* (EHEC) to attach and efface HeLa epithelial cells. Indeed, in the presence of aurodox a 36 % reduction of infection and effacing lesions was observed, resulting in a >3 log reduction in EHEC colonization. Whole-transcriptome analysis revealed that aurodox inhibits the expression of numerous virulence genes, including the locus of enterocyte effacement (LEE) pathogenicity island. Aurodox also downregulates the expression of *ler*, a master regulator of T3SS. Conversely, McHugh *et al.* demonstrated that inhibition of the TS33 by aurodox could be overcome by overexpression of *ler*. Finally, it was shown that aurodox does not trigger the EHEC SOS response or induce Shiga toxin expression in *C. rodentium.* The SOS response and subsequent expression of shiga toxin in EHEC cases can lead to life-threatening haemolytic uremic syndrome. Therefore, it is clinically relevant that aurodox can inhibit the TS33 without inducing these responses [36].

Although the exact target of aurodox remains to be elucidated, this work highlights its potential as a clinical anti-virulence compound. In the context of the current antimicrobial resistance crisis [39], the development of novel strategies to combat infection is paramount, with the discovery and characterization of anti-virulence compounds being one of these strategies. It is very possible that resistance to T3SS suppression has already evolved, and thus worthwhile to carry out further investigation into the mechanism of *ler* repression by aurodox, the possible mechanisms by which resistance to aurodox could arise, and the evolution and chemistry of aurodox biosynthesis [36].

This work by McHugh *et al.* demonstrates the value of reinspecting molecules that may be non-viable as clinical antimicrobial agents alongside the methodology for the complex task of characterizing mechanisms of virulence inhibition.

#### Phylogenetic reconciliation reveals the natural history of glycopeptide antibiotic biosynthesis and resistance [40]

With an increase in the quantity of publicly available microbial genomic data, numerous bioinformatic tools have been developed to help us understand gene functions and mine for novel BGCs [41]. These genomic data can also be leveraged to study the phylogenetics of biosynthetic genes, to give insights into how particular natural product pathways might have evolved.

A study by Waglechner *et al.* demonstrated the use of phylogenetic reconciliation in order to understand the origin and evolution of glycopeptide antibiotics (GPAs), a class of actinomycete natural product that includes last-line-of-defence antibiotics such as vancomycin and teicoplanin [40].

GPAs are glycosylated peptides synthesized by non-ribosomal peptide synthetases (NRPS). They target cell wall synthesis of Gram-positive bacteria by binding to the acyl-d-alanyl-d-alanine terminus of the growing peptidoglycan chain. GPAs share a common heptapeptide scaffold containing aromatic amino acids that are subject to extensive oxidative crosslinking. Further tailoring reactions are carried out after release from the NRPS machinery, to introduce sugar residues, chlorination and lipid chains [42, 43]. Alongside these tailoring enzymes, GPA BGCs also include genes for resistance, regulation and export. As these features are not conserved in all GPA BGCs, their evolutionary roles are difficult to study in isolation, but comparative analysis of several BGCs can provide reconstruction of the natural history of GPAs [40].

Waglechner *et al.* compared 71 GPA BGCs from 8 genera of *Actinobacteria *, which were used to construct multiple species phylogenies. The phylogeny of each GPA gene and domain was separately compared with a dated species tree to identify evolutionary milestones of GPA BGCs using phylogenetic reconciliation [40].

This approach showed that GPA precursor biosynthesis, as well as ancestors of export and regulation, are the most ancient components of GPA BGCs, at over 1 billion years old. This includes enzymes responsible for the generation of non-proteinogenic amino acid precursors, such as 3-deoxy-d-arabinoheptulosonate 7-phosphate synthase (DAHPS), chorismate mutase (CM) and prephenate dehydrogenase (Pdh), which therefore predate their incorporation into GPA BGCs. The fact that these precursors are older than other pathway components suggests that GPA BGCs arose from a pre-existing pool of genes [40].

In contrast, resistance is relatively contemporary. Most GPA BGCs contain the *vanHAX* genes for self-resistance, and the reconciled roots of these genes map from 140 million years ago (Ma) for *vanA* to 404 Ma for *vanH* and *vanX*. Specific tailoring enzymes also have some of the youngest reconciled dates: the crosslinking P450 enzymes OxyA, C and E arose 202 Ma within *Nonomuraea *, along with acylation; glycosylation arose 224 Ma and halogenation arose 731 Ma within *Amycolatopsis *. The structurally central heptapeptide scaffold was also shown to arise 300–500 Ma [40].

Overall, this work puts antibiotic biosynthesis and resistance into an evolutionary context, and builds on previous studies that were limited to phylogenies of specific marker genes [44, 45]. The study of GPA evolution gives valuable insights into how new molecules arise in nature, and is also important for guiding bioengineering approaches for the production and discovery of potential new antibiotics in the future [45, 46].

These studies highlight a diverse range of methodologies that can be applied to identify new antibiotics and explore the clinical potential of known natural products. From isolating novel bacteria from unique environments, investigating the activities of known molecules and learning biosynthetic lessons from evolution, combined these approaches can help uncover the wide-ranging functions of metabolites produced by actinomycetes, and how these molecules are biosynthesized.

### Ecology and host interactions

Actinomycetes are an ecologically diverse group, able to occupy a huge range of environments and niches. This is exemplified by the breadth of habitats actinomycetes were detected in and isolated from in 2019, ranging from marine sediments in the Arctic and Antarctic [47] to tropical mangrove sediments in India [48]. They have been found in wetlands [49], hyper-arid Atacaman desert soils [50], underground in Shuanghe cave systems [51] and in Himalayan mountain pine forests [52]. In addition, actinomycetes are frequently found associated with a host organism, forming symbiotic relationships with insects such as bees [53], fungus-farming ants and pine beetles [54]. They can also be associated with plants, ranging from the economically important cereal crop wheat (*Triticum aestivum*, 53) to the Japanese black pine tree [55]. The varied ecology of actinomycetes provides a deep well of biological intrigue and has led to important discoveries such as new antibiotics [47, 54] and agricultural biocontrol applications [56, 57].

#### Awakening ancient polar *Actinobacteria *: diversity, evolution and specialized metabolite potential [47]

Actinomycetes produce a plethora of biologically active and chemically diverse metabolites that provide over two-thirds of antibiotics. Marine polar and sub-polar environments, which have been largely understudied due to their extreme climate, may yield previously undiscovered actinomycetes with novel antimicrobial metabolites. Indeed, 454 sequencing of bacteria from Arctic sediment samples found that 10 % of the bacterial community were *Actinobacteria* [58]. These ecosystems are highly vulnerable to global climate change and so exploration of the polar sediment microbiome may provide insights into the biomass, diversity and community composition of microbial communities in fluctuating environments. Furthermore, it has been proposed that geographical isolation may determine bacterial population structure, and investigation into the relationship between community composition, functional genes and sediment depth may elucidate more about actinomycete evolution and ecology [47].

This study, performed by Millán-Aguiñaga *et al.*, attempted to isolate rare actinomycetes from sediment cores retrieved from Artic and sub-Artic environments using both culture-dependent and metagenomic investigation in order to isolate specialized metabolite production. The study obtained 12 previously undisturbed sediment cores sourced from both the Antarctic and the Artic in a manner that endeavoured to preserve the sediment–water interface and consistent 4 °C temperature. These sediment cores were anaerobic, whilst the 50 actinomycete-like strains isolated from the sediment were cultured aerobically, suggesting that spores may be metabolically inactive. Of the isolated strains, 78 % belonged to the phylum *Actinobacteria *, with some rare actinomycetes, including 25 Antarctic *Pseudonocardia* strains. Sequencing of the 16S rRNA gene and phylogenetic analysis demonstrated that KRD-291, 1 of the 25 *Pseudonocardia* strains isolated, was related to *Pseudonocardia sediminis,* a species previously isolated from South China Sea sediment [59]. The remaining *Pseudonocardia* isolates were most closely related to the type strains of *Pseudonocardia petrolephila* and *Pseudonocardia serianimatus,* but sequence similarity values suggest that many may represent novel species. Radiocarbon dating of one core, the Artic sediment core GC067, dated it at between 8287 (upper) and 26 000 (lower) years old. From this, three genera of *Actinobacteria* were isolated, *Halomonas *, *Microbacterium* and *Rhodococcus.* Additionally, more potentially novel species were isolated from an Antarctic core, belonging to the genera *Agrococcus* and *Salinibacterium *. Following DNA extraction, MinION sequencing was performed for metagenomic analysis in order to investigate the bacterial diversity within the cores. This revealed that bacterial reads comprised between 1–5 % of total reads and *Actinobacteria *, including *Microbacterium,* were found to be present in both sediment cores, but in higher in abundance within Artic sediment [47].

To investigate the biochemical potential of the isolates, 15 strains belonging to the genera *Pseudonocardia *, *Microbacterium *, *Agrococcus *, *Rhodococcus* and *Pseudomonas* were chosen based on location and phylogeny. The strains were screened for bioactivity against both Gram-positive and Gram-negative bacterial pathogens. Although no antimicrobial activity was detected, analysis of the tandem mass spectrometry (MS/MS) data at the genus level resulted in a molecular network comprising 1652 nodes. Further investigation into the *Pseudonocardia* strains isolated from the upper, middle and lower sections of sediment core BC043 revealed considerable chemical diversity across core depth, and therefore time, suggesting that sediment depth influences chemical diversity [47].

The study concludes that polar bacteria may have considerable chemical potential, with the biochemical profile of these isolates suggesting that many of these metabolites may be novel natural products.

#### Local adaptation of bacterial symbionts within a geographic mosaic of antibiotic coevolution [60]

In addition to extreme and unexplored habitats, novel antibiotics can be found in host-associated systems [5]. For example, the *Pseudonocardia *-dominated actinomycete community associated with fungus-farming leafcutter ants produce a range of antifungal and antibacterial compounds, such as the antifungal nystatin P1 [61]. Fungus-farming ants depend entirely on their fungus garden (*Leucoagaricus gongylophorus)* as a source of food. This fungus can be susceptible to infection by specialized fungal pathogens from the genus *Escovopsis*. By utilizing the antifungal secondary metabolism of their actinomycete symbionts, the ants can protect the fungus garden from *Escovopsis* infection. Other ant species have also been shown to cultivate fungus, including the African plant ant *Tetraponera penzigi*, and the exploration of this symbiosis led to the discovery of an entirely new class of antibiotic compounds called formicamycins, demonstrating the potential contained within these unique ecological interactions [62].

It is thought that fungus-farming ants have been challenged by *Escovopsis* infections for much of the ~55 million years that they have been cultivating *L. gongylophorus*, and have likely been utilizing the antifungal capabilities of actinomycete symbionts for millions of years [63]. Despite this, little is known about the coevolutionary history of this symbiosis. Caldera *et al.* aimed to place this system within an evolutionary framework by testing whether the coevolution occurring between the *Pseudonocardia* symbiont and the *Escovopsis* nest pathogen fits the geographic mosaic theory of coevolution (GMC). The GMC framework suggests that coevolution will vary among different populations of leafcutter ants depending on local geography and ecology. Previous work from the group showed that the extent of *Escovopsis* inhibition *in vitro* is proportional to the ability of *Pseudonocardia* to suppress the disease *in vivo* [64]. Using this principle, they tested the ability of 52 *Pseudonocardia* strains (isolated from different ant colonies across Central America) to inhibit different strains of *Escovopsis*. They found greater inhibition of an *Escovopsis* strain correlated with its abundance in leafcutter ant nests, indicating that *Pseudonocardia* spp. are generally more adapted to suppress the most common strains of the pathogen.

To understand how well adapted *Pseudonocardia* strains are, Caldera *et al.* conducted a matrix bioassay, consisting of 2417 individual bioassays challenging different *Pseudonocardia* isolates from the Panama Canal region with *Escovopsis* strains isolated from different sites. The Panamanian canal isolates were less capable of inhibiting *Escovopsis* strains from Costa Rica or from a distant region of Panama. Interestingly, they were also worse at inhibiting *Escovopsis* strains from Barro Colorado Island (BCI), an island formed around 100 years ago when the Panama Canal was flooded. Further analysis confirmed that the BCI population is, in fact, locally adapted, whereas local adaptation was less present in some of the surrounding mainland populations. This variation in the extent of local adaptation is consistent with the GMC framework.

Lastly, they performed genomic analysis using both published data and whole-genome sequencing. This revealed that the population of *Pseudonocardia* symbionts and *Escovopsis* strains on BCI is, in fact, genetically distinct. Using the 29 *Pseudonocardia* genomes they produced, they were also able to correlate the presence or absence of certain BGCs with the ability or inability to inhibit different *Escovopsis* strains. For example, BCI *Pseudonocardia* have gained a unique bacteriocin cluster, which is likely used to inhibit other actinomycete strains attempting to colonize the ants. They have also acquired a mutation in a gene within an NRPS BGC. This shows that horizontal gene transfer is not the only mode by which *Pseudonocardia* spp. can adapt to combat *Escovopsis* strains, and that selection can still favour beneficial genomic mutations.

Overall, this study takes a comprehensive look at the evolutionary dynamics governing the interaction between symbiotic *Pseudonocardia* strains and the *Escovopsis* nest pathogen. They manage, for the first time, to apply the GMC framework to a microbial population in order to assess local coadaptation between two microbes. This is a valuable way to understand how these populations have adapted and has revealed that populations that are more isolated can carry distinct BGCs. This knowledge could help guide isolation approaches in the future to maximize the likelihood of finding novel antibiotics.

#### A mutualistic interaction between *Streptomyces* bacteria, strawberry plants and pollinating bees [65]

We have long known that actinomycetes, such as streptomycetes, can occupy plant-associated niches [66] in addition to insect-associated niches [67], and that they can protect either host organism from disease [68, 69]. However, questions remain surrounding the exact nature of the relationship. Are streptomycete endophytes mobile within plant tissues? Do entophytic streptomycetes (those that can grow within plant tissues) influence multi-trophic interactions involving the host?

Kim *et al.* [65] begin to address these questions as they describe a mutualistic tri-partite interaction between *Streptomyces* spp., strawberry plants (*Fragaria* x *ananassa*) and honeybees. Using 454 16S rRNA gene pyrosequencing, they consistently found streptomycetes within the flowers and pollen of strawberry plants during early life. In later life, however, the abundance of streptomycetes falls, a change that correlated strongly with the onset of grey mould disease (*Botrytis cinerea*). Interestingly, they were able to combat grey mould disease successfully via a topical application of streptomycete strains isolated from the flowers of strawberry plants. Typically, streptomycetes colonize the roots [66] as plants acquire most of their beneficial microbiota horizontally from the soil community [70]. Isolation of *Streptomyces* species from aerial plant tissues (the phyllosphere) raised a question – how do streptomycetes migrate from the soil to the flowers? To address this, Kim *et al.* used both qPCR and fluorescence microscopy to show for the first time that *Streptomyces* spp. isolates are able to migrate from the roots, via the xylem, to colonize the stamen and the pollen granules. This supports a model whereby streptomycetes colonize the roots from the soil, and then migrate via the xylem to colonize the phyllosphere.

Honeybees in contact with streptomycete-containing pollen granules were also colonized. The honeybees benefit from this as bees fed pollen containing streptomycete isolates were significantly more resilient to infection by two bacterial entomopathogens (* Paenibacillus larvae* and *Serratia marcescens *). Remarkably, Kim *et al.* also showed that bees can transmit *Streptomyces* spp. between plants. In a single chamber, three separate groups of strawberry plants were grown; one inoculated with *Streptomyces* bacteria and two uninoculated. They capped the flowers of one uninoculated group, blocking honeybee access. After honeybees fed from these strawberry flowers streptomycete isolates could be detected in their guts. Additionally, the isolates could now be detected in the flowers of the uncapped, uninoculated plants. No *Streptomyces* spp. were found in the uninoculated capped plants, showing that where honeybees had access, they were able to transmit *Streptomyces* bacteria flower to flower.

In short, Kim *et al.* were able to provide insights into a multi-partite interaction between streptomycetes, strawberry plants and honeybees. They showed that streptomycete colonize strawberry roots and migrate via the xylem to the flowers. Here they colonize pollen granules and in turn honeybees consuming this pollen are colonized by streptomycetes. As the bees travel between flowers, they transmit *Streptomyces* spp*.* between plants. All the while, streptomycetes provide both their plant and insect hosts with protection from disease. This study is among the first to demonstrate how *Streptomyces* spp. are able to migrate within a plant host and to observe the transmission of endophytic *Streptomyces* species among different hosts. Many questions remain. Which secondary metabolites are responsible for protection of the host against fungal and bacterial pathogens? What plant signals govern recruitment of streptomycetes, and by what mechanism are the bacteria able to traverse the xylem? Is bee gut colonization transient, or do the insects acquire *Streptomyces* spp. long term? While much remains to be investigated, Kim *et al.* have provided compelling evidence to support the idea that streptomycetes can colonize the phyllosphere and have highlighted a fascinating multi-partite interaction. The symbiotic relationship between pollinating insects and flowering plants is a keystone interaction of crucial ecological importance [71], and this study indicates that symbiotic actinomycetes may play an important role in this long-studied process.

From the deep sediments of the poles, through the jungles of Central America, to the flowers of a strawberry plant and the gut of the honeybee, these publications exemplify the breadth of habitats for which actinomycetes are adapted. In all these cases actinomycetes produced antibiotics, showing the breadth of the potential these organisms possess for the discovery of novel antibiotics from natural ecosystems, further highlighting the importance of preserving these environments. As shown through the host-associated systems, actinomycetes can be significant ecological partners in these ecosystems, displaying intricate co-adaptation with either the host or the pathogen.

### Technology and methodology

Molecular manipulation of actinomycetes is challenging, with even the most basic techniques being more laborious and time-consuming than in many other commonly studied micro-organisms [72]. There are many useful and powerful tools available, or under development, for actinomycete research. However, a significant amount of contemporary work has become underpinned by two technologies. The first is long-read, next-generation whole-genome sequencing (WGS), such as PacBio and, to a lesser, but ever-increasing extent, Oxford Nanopore, which has allowed the accurate construction of genomes rich in GC-repetitive regions [9, 73]. The second is CRISPR/Cas9-based genome editing technologies, the first of which, pCRISPomyces-2, was introduced in 2015 and has allowed for precise, scar-free genome modifications, deletions and replacements [74].

#### antiSMASH 5.0: updates to the secondary metabolite genome mining pipeline [75]

As mentioned throughout this review, one of the key features of actinomycetes are the numerous BGCs in their genomes encoding for the biosynthesis of a plethora of specialized metabolites. Identification of these clusters has become an essential part of the workflow for the discovery of novel compounds and in guiding biological and chemical characterization [76]. First released in 2011, the antibiotics and secondary metabolites analysis shell (antiSMASH) has revolutionized the ease of BGC *in silico* identification [77]. This software, available as a web-based application and a standalone tool, is capable of identifying and putatively classifying BGCs from genome sequences provided or within its own database. Iterations have since added support for independent tools, including the CRISPR sgDNA design software CRISPY-web and the Antibiotic Resistance Target Seeker (ARTS) [78–80]. The most recent update, antiSMASH 5.0, introduces numerous new features. The recommended mode for BGC detection by antiSMASH is through conserved core enzyme co-occurrence identification. In antiSMASH this is underpinned by gene cluster rules, which are manually curated and validated based upon hidden Markov models (HMMs) derived from multiple databases, including the Pfam protein family database [75, 81]. Version 5.0 introduces new rules and revises current rules to allow the identification of 8 new biosynthetic types, bringing the total up to 52. These new BGC classes include β-lactones, pseudopyronines, radical S-adenosylmethionine-associated RiPPs and fungal RiPPs. Improvements have been made to type II polyketide synthase (PKS) analysis, including prediction of starter unit, elongation cycles number and patterns [75].

It is a common occurrence for distinct BGCs to be encoded adjacent to each other, especially in actinomycete plasmids where numerous clusters can be found side by side. Historically, antiSMASH has had issues with identifying where one BGC ends and another starts, with such circumstances often necessitating manual curation of results to separate these misconcatenated clusters. Version 5.0 introduces new terminology to better describe results, for example to distinguish between neighbouring and true chemical hybrid clusters [75].

In addition to this, antiSMASH 5.0 has undergone a user interface (UI) redesign and refactored its entire code, migrating from Python 2.7 to Python 3.5–3.7 for longevity, stability and subsequently speed. Historically, results would be delivered after several hours, and this has been reduced by 4–11×, leading to job completion within 40 min for a typical submission. The UI modernization has increased the accessibility of the results pages, delivering greater detail and interaction capabilities. For example, it is now possible to select BGC ‘functional’ units such as the core biosynthetic enzymes [75]. To newcomers actinomycete research can seem intimidating, thus it is vital to lower these walls as a community. The creators of antiSMASH have taken this and other feedback into account, removing the often misinterpreted ClusterFinder algorithm from the online version. It is, however, still available in the more exhaustive downloadable version [75].

It may seem unusual to feature a software version update publication, but antiSMASH has been pivotal to actinomycete secondary metabolism research. Each iteration is a step towards a comprehensive, and accessible, BGC identification and classification tool and thus is featured in the hope of highlighting this tool for the wider microbiology community.

#### Highly efficient DSB-free base editing for streptomycetes with CRISPR-BEST [82]

The transition from PCR-targeted gene replacement techniques such as ReDIRECT to contemporary CRISPR-based systems has simplified and streamlined *in situ* manipulation of actinomycete genomes [74, 82, 83]. The second featured publication for the technology and methodology section introduces a new CRISPR-based tool in response to issues arising from Cas9-containing systems such as pCRISPomyces-2 [74, 82]. The expression of the Cas9 enzyme, the dual RNA-guided DNA endonuclease used to create the double-stranded break (DSB), which facilitates the chromosomal rearrangements, can be toxic to many actinomycetes, leading to undesirable side-effects [84]. The linear nature of some actinomycete chromosomes tends to allow the target to withstand relatively large rearrangements and deletions, especially in arm regions of the chromosome, a problem faced by Tong *et al.* while trying to delete the duplicated kirromycin biosynthetic pathways in *Streptomyces collinus* Tü365 [82, 85].

*S. collinus* Tü365 encodes the 82 kbp duplicated pathway for this BGC in either arm of the chromosome, but deletion of a speculated core biosynthetic gene (*kirN*) from one copy of the BGC using the classical Cas9-based system led to 788 and 630 kb deletions in both the left and right chromosomal arms, respectively. In addition, it led to complete loss of kirromycin production rather than the intended knockdown, as both copies of the BGC, and many off-target genes, had been lost due to genomic instability resulting from the Cas9-induced DSB [82].

To overcome this, Tong *et al.* develop a new DSB-free CRISPR system that utilizes a deaminase-based editor for single-nucleotide resolution editing in actinomycetes and only requires a 20 nucleotide protospacer to guide editing. This CRISPR-Base Editing System (CRISPR-BEST) comes in two forms: CRISPR-cBEST, which converts C : G to T : A and CRISPR-aBEST for A : T to G : C. Cytidine-to-thymidine mutations allow the introduction of STOP codons, whilst loss-of-function mutations can be induced by single-nucleotide mutations to guanosine [82, 86]. Deaminase-based editing systems do not require a DSB; deamination can only occur in single-stranded DNA and this restriction limits the base pair conversion to the nicked strand in the Cas9n : sgRNA : target R-loop region [82, 87]. Similar systems have been used with success in human cell line editing because these features allow specific editing with significantly less chance of off-target effects [88, 89].

These new tools were validated in the model organism *S. coelicolor* A3(2) and the base pair editing efficiency conversion of cytidine to thymidine was proven to be the most efficient of the four possibilities for CRSIPR-cBEST (TC>CC>AC>GC) and thymidine to cytidine for CRISPR-aBEST (TA>GA>AA>CA). Off-target effects were quantified using WGS with single-nucleotide polymorphisms (SNPs) being mapped to determine meaningful amino acid changes. For 2 CRISPR-cBEST strains 34 and 24 meaningful mutations were identified (outside the desired effect), whilst only 21 and 20 were identified for the 2 CRISPR-aBEST-edited strains. Further validation was performed by successfully introducing STOP codons into key enzymes from four representative BGCs in *Streptomyces griseofuscus* before the system was used to tackle the *kirN* problem in *S. collinus* Tü365 [82].

Using CRISPy-web (https://crispy.secondarymetabolites.org), which has been updated to be compatible with CRISPR-BEST protospacer design, a STOP codon was incorporated into both copies of *kirN*, leading to reduced kirromycin biosynthesis and thus reduced inhibitory zones in bioactivity assays. The reduced yield of kirromycin was confirmed using LC-MS [81]. For the final plasmid delivery system (available at https://www.addgene.org/131464/), a Csy4-based sgRNA self-processing system was included to aid simple multiplex editing. Previous systems required individual promoter and terminators for every sgRNA included, but the use of Csy4 allows for the incorporation of multiple snRNAs under a single promoter and terminator [82, 90].

**Fig. 2.**
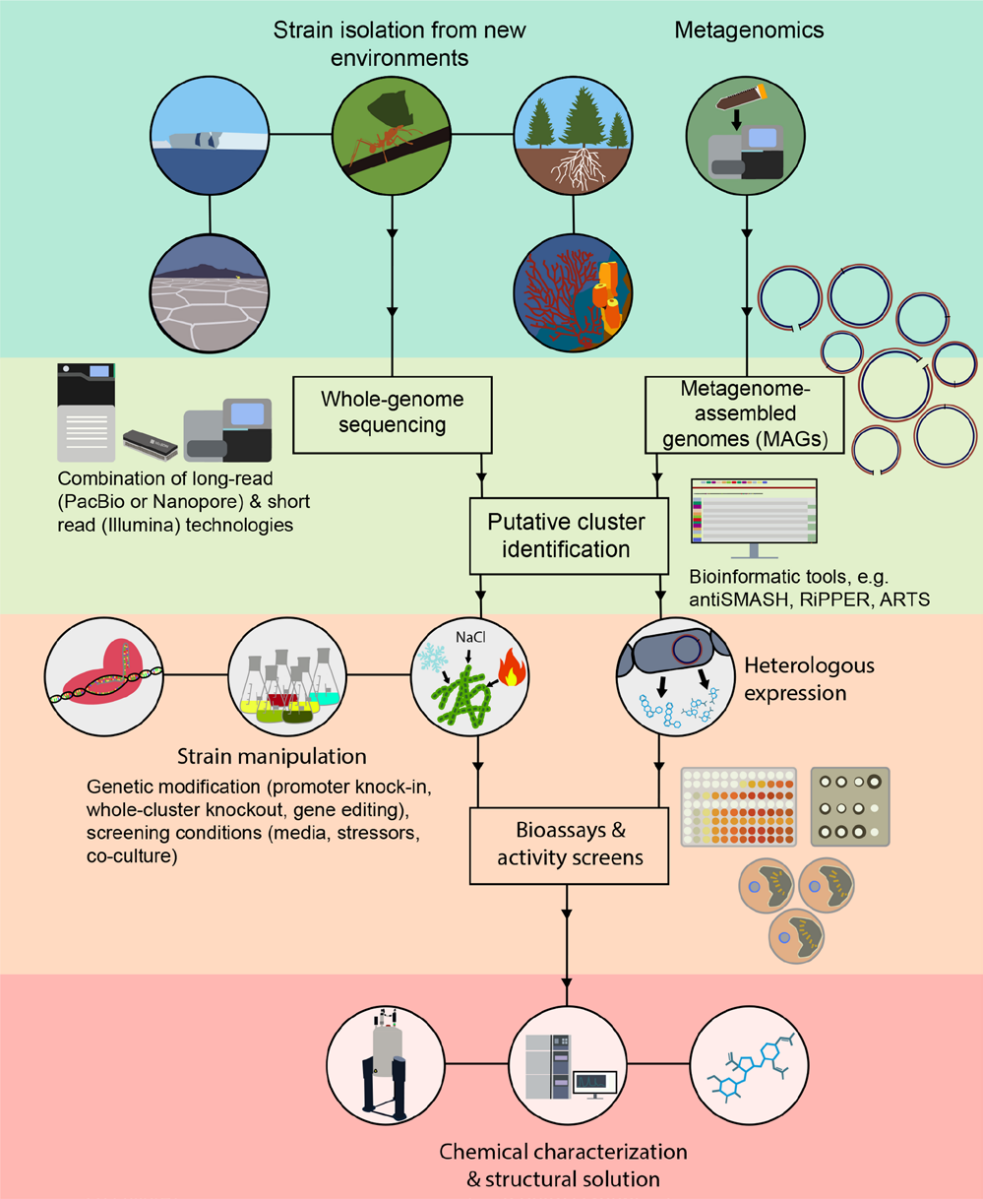
Graphic showing the general antibiotic compound discovery pipeline. The process usually begins with the isolation of novel strains or DNA from environmental samples. Once genomes and metagenomes are sequenced and assembled, the sequences can be analysed with tools such as RiPPER, antiSMASH or ARTS to identify biosynthetic gene clusters (BGCs). A combination of cluster synthesis, cloning and heterologous expression, or strain manipulation through CRISPR or varied conditions, can then be used to encourage the production of novel antibiotics. The production of antibiotics is screened for using bioactivity assays. Compounds can then be further characterized using techniques such as NMR or mass spectrometry.

Technology and methodology are the basis of modern research and when dealing with fastidious micro-organisms such as actinomycetes even small advances can significantly ameliorate day-to-day work. You can see in Fig. 2 that the processes here not only complement each other but also enhance the standard workflow. At the time of writing, it remains to be seen what 2020 brings in terms of technological and methodological advancements for actinomycete research, but it is truly exciting to see the outcomes of tools presented in 2019.

## Future prospects

Recent advances in genomic sequencing as well as the development of more sophisticated genome mining and bioinformatic tools mean that there is a promising future for natural product discovery from actinomycetes. It has long been known that >99 % of bacteria cannot be cultivated [91] and that the cultivation of actinomycetes remains a challenge, especially for rarer and highly specialized strains. One approach to overcome this is the utilization of novel isolation devices such as the ichip, which has been shown to increase microbial recovery from 5- to 300-fold [92]. Alternatively, cultivation can be circumnavigated by using metagenomics [93]. This culture-independent approach can enable researchers to recover entire genomes from the environment. In combination with antiSMASH and other recently developed tools [31, 80, 94], this approach could identify novel putative BGCs from unculturable microbes, which could then be expressed heterologously in a model host organism [95]. The development of databases such as Global Natural Products Social Molecular Networking (GNPS) then allows for in-depth analysis of microbial metabolomic data to aid discovery [96].

Combined, these approaches have the potential to help revitalize antibiotic discovery in the future, which in turn drives the investigation into other key areas of actinomycete research, including developmental biology, ecological interactions and methodology expansion.
